# Through the Eye: Retinal Changes of Prenatal Mercury Exposure in Grassy Narrows First Nation, Canada

**DOI:** 10.3390/ijerph23010001

**Published:** 2025-12-19

**Authors:** Véronique Small, Aline Philibert, Annie Chatillon, Judy Da Silva, Myriam Fillion, Donna Mergler, Benoit Tousignant

**Affiliations:** 1 School of Optometry, Université de Montréal, 3744 Jean-Brillant, Montreal, QC H3T 1P1, Canada; 2 Department of Social and Preventive Medicine, School of Public Health, Université de Montréal, Montreal, QC H3N 1X9, Canada; 3 Centre de Recherche Interdisciplinaire sur le Bien-Être, la Santé, la Société et L’environnement (CINBIOSE), Université du Québec à Montréal, Montréal, QC H3C 3P8, Canada; 4 Grassy Narrows First Nation, Grassy Narrows, ON P0X 1B0, Canada; 5 Département Science et Technologie, Université TÉLUQ, 5800, Rue Saint-Denis, Bureau 1105, Montréal, QC H2S 3L5, Canada

**Keywords:** Indigenous people, First Nations, methylmercury, prenatal mercury exposure, optical coherence tomography, retina, Canada

## Abstract

Since the 1960s, Grassy Narrows First Nation, Canada, has been exposed to methylmercury (MeHg) from fish consumption following Hg discharge from a chloralkali plant. Prenatal exposure to MeHg is known to affect the neurodevelopment of fetuses and the retina is sensitive to neurodevelopmental damage. The multidisciplinary, cross-sectional Niibin study, developed with Grassy Narrows First Nations, included visual examinations with retinal evaluation using optical coherence tomography (OCT). The present analyses focused on the 59 participants (116 eyes) with umbilical cord Hg measurements, sampled between 1971 and 1992. Associations between cord blood Hg and retinal thickness layers surrounding the optic nerve head (RNFL) and inner macula (GC-IPL) were examined using mixed-effect models. Higher cord blood Hg was significantly associated with reduced thickness of GC-IPL layers across all macular sectors; less pronounced associations were observed for RNFL. A qualitative clinical assessment of the OCT results showed that persons with cord blood Hg concentrations ≥ 5.8 µg/L were more likely to present bilateral abnormal retinal thinning (OR = 3.51; [95% CI: 1.06–11.53]). These findings suggest that, in this Indigenous community, prenatal MeHg exposure may have enduring effects on retinal thickness and underline the importance of OCT technology in providing tailored eye care.

## 1. Introduction

The people of Asubpeeschoseewagong Anishinabek (Grassy Narrows First Nation) have been profoundly affected by an ecological disaster [1]. Between 1962 and 1975, a chloralkali plant of a pulp mill in Northwestern Ontario, Canada, discharged nearly ten tons of mercury (Hg) into the Wabigoon–English river system, which flows into Grassy Narrows’ traditional territory [2]. When inorganic Hg is introduced into organically rich aquatic systems, it undergoes a transformation into methylmercury (MeHg), which then bioaccumulates in organisms and biomagnifies through the aquatic food chain, making up 95% of the Hg content in fish [2,3]. In 1968, Hg concentrations in walleye downriver of the plant were between 2.75 μg/g and 19.6 μg/g [4]. While fish consumption is vital for the nutritional and identity security of Indigenous people generally, it was even more so for this community for whom walleye (*Sander vitreus*), a freshwater high-end predator, was a dietary mainstay as well as an important economic driver, with the majority of community members working as fishing guides or involved in commercial fishing [1,5,6,7]. Controls on Hg emissions were ordered in 1970, and the plant’s Hg cell process was officially closed in 1975 [2]. Concentrations of Hg in fish decreased and stabilized by the end of the 1980s [2]. In this region, walleye still have the highest Hg concentrations in Ontario and in Canada [2,8,9].

Between 1970 and 1997, government biomonitoring programs assessed Hg concentrations in blood, hair, and umbilical cord blood of people from Grassy Narrows [7]. Blood and hair samples, analyzed for the composition of the inorganic and organic components, showed that MeHg made up over 75% of total Hg [10,11]. The evolution of Hg concentrations in human samples paralleled those observed in fish, with the highest concentrations in the first years of sampling and decreasing to a plateau in the early nineties [12]. Recent research, carried out in collaboration with the community, has identified significant adverse effects associated with past long-term Hg exposure, including persistent symptoms of nervous system dysfunction [13,14], cognitive loss [15], and visual impairments, such as constricted visual fields [16,17].

Prenatal MeHg exposure is known to be particularly toxic [18,19]. MeHg is actively transported across the placenta and constitutes 95% of umbilical cord blood Hg [20,21,22,23,24]. The infant blood–brain barrier is not fully developed until approximately six months after birth [25,26]. MeHg may impact neural function by disrupting neurotransmission, oxidative homeostasis, and cellular integrity [27,28]. The most severe form of prenatal MeHg poisoning was first identified as congenital Minamata disease [29,30]; children exposed prenatally often experienced more severe neurological symptoms than their mothers [30,31,32]. Follow-up studies of patients between 50 and 64 years of age with congenital Minamata disease show accelerated functional loss [33].

While there are many studies on the impact of prenatal MeHg exposure on children’s and young adults’ neurodevelopment [34,35,36], few studies have focused on the impact of prenatal Hg exposure on the visual system [37]. Studies of children prenatally exposed to MeHg in the Faroe Islands have shown effects on visual evoked potential (VEP) latency, indicating disruptions in brain processing of visual stimuli [38,39]. Similar findings were reported in a cohort of preschool and school-aged Inuit children in Canada [40]; a five-year follow-up showed that the effect was persistent over this period [41]. Methylmercury-related changes in VEP were likewise observed in school-aged children in Japan, but only for boys [42].

While VEPs assess the integrity of the visual pathway from the retina to the visual cortex [43], little is known about the effect of MeHg on ocular structures. The retina, an extension of the brain, shares its embryonic origin with the central nervous system (CNS), with retinal ganglion cells having similar properties to other CNS neurons [44]. In animal models, studies show MeHg accumulation in retinal layers [45,46]. In zebrafish larvae, MeHg toxicity interferes with retinogenesis by contributing to cell apoptosis [47], and by affecting the expression of specific genes, decreasing the number of GABAergic neurons in the neuroretina [48,49]. As the retina is often seen as the window to the CNS, high-resolution imaging, like optical coherence tomography (OCT), provides visualization of retinal layers in a fast and non-invasive way [50]. By segmenting the different layers of the retina and optic nerve, OCT improves detection of abnormalities and clinical management [50,51,52].

The objective of the present study was to use OCT to describe current retinal architecture in adults of Grassy Narrows First Nation with respect to umbilical cord blood Hg concentrations.

## 2. Materials and Methods

The present study is part of the Niibin Project, conducted in partnership with Grassy Narrows First Nation. The project adheres to the First Nations Principles of Ownership, Control, Access and Possession of OCAP^®^ [53]. OCAP^®^ is a registered trademark of the First Nations Information Governance Centre (FNIGC) [53]. The Niibin project uses a participatory and integrated knowledge approach to document health impacts of the community’s exposure to Hg [12,13,14,15,16,17,54].

### 2.1. Prenatal Hg Exposure

Between 1970 and 1992, a government biomonitoring program collected umbilical cords from Grassy Narrows women who gave birth at the local hospital for Hg analysis [7]. The analytical methods for Hg are described elsewhere [10,11,55]. The community of Grassy Narrows repatriated the data from the First Nations and Inuit Health Branch of Indigenous Services Canada and the Ontario Ministry of Health and Long-Term Care and shared it with the authors. All samples were analyzed for total Hg [11,55]; Hg concentrations for 211 umbilical cords from Grassy Narrows were recovered and included in a database with year of birth and sex [12]. Although cord blood MeHg accounts for at least 95% of total Hg [20], we prefer to use Hg when referring to the actual measurements and MeHg when discussing the effects.

### 2.2. Visual Examination

Visual examinations were performed in the summers of 2021 and 2022 by three research optometrists at Sakatcheway Anishinaabe School in Grassy Narrows in a classroom converted into an examination room [16]. They included visual acuity measurement, electronic autorefraction, non-mydriatic slit lamp assessment and cataract grading, color vision and contrast sensitivity assessment, automated visual field, and OCT. The full protocol can be found elsewhere [16]. OCT, visual acuity, and autorefraction were used for the present analyses.

### 2.3. Distance Visual Acuity

Distance visual acuity was measured using an LED monitor and the Early Treatment Diabetic Retinopathy Study (ETDRS) chart in logMAR [56]. Participants used their best optical correction when available. The measurements were monocular and pinhole visual acuity was performed in cases where a visual acuity measurement was 0.3 logMAR or higher [16].

### 2.4. Autorefraction

Ametropia was measured using the HandyRef-K autorefractometer (NIDEK CO., Ltd., Gamagori, Japan) to evaluate the refractive value of each participant’s eye [16]. Spherical power characterizes ametropia, distinguishing myopia from hyperopia and their potential effects on retinal thickness as a proxy to the axial length of the eye [57,58].

### 2.5. Optical Coherence Tomography (OCT)

Two types of scans were performed. The optic disc cube (200 × 200) protocol measures the retinal nerve fiber layers (RNFL), which includes average thickness and the thickness of the RNFL values for each quadrant of the optic nerve (superior, inferior, nasal, temporal). These values express the thickness of the entirety of retinal layers converging to the optic nerve head, divided by quadrants and with the average value [59,60,61]. The macular cube protocol (128 × 128) offers a qualitative and quantitative evaluation of the ganglion cell layers and the inner plexiform layer (GC-IPL) representing the ganglion cells of the macula, the most central and sensitive part of the retina used in vision [62]. The sector map divides the ring thickness map into six sectors excluding the foveolar region, with measures for average and minimal GC-IPL thickness [60,61].

OCT measurements were collected using the Cirrus HD-OCT (Carl Zeiss, Meditec Inc., Oberkochen, Germany). The data acquired are compared to a normative database using an algorithm specific to the manufacturer [61,63]. The optic nerve protocol database contains 284 subjects (mean age 46.5 years; 19–84 years) and the macula protocol database contains 282 subjects (mean age 46.6 years; 19–84 years). Refractive errors of the database are limited to ametropia between −12.00D and +8.00D. There is a mix of ethnicities in the database. The information provided by the instruments are adjusted only for age [60].

Device measurements are presented numerically in microns and categorized using color-coded age adjusted normative data provided by the manufacturer. The normal range includes the thickest values (>95th percentile), coded white, while green represents thicknesses within the 5th to 95th percentile. The thinnest measures, classified as abnormal for their age, are yellow (1st < 5th percentile) and red (<1st percentile).

OCT scans were reviewed by three optometrists (VS, AC, BT) for reliability, clinical interpretation, and the detection of ocular pathologies. Post hoc exclusions identified during the preliminary examination of the results consisted of OCT signal < 6/10, significant measurement artifacts, the presence of pathology preventing imaging (cataracts, corneal opacity), and the presence of evident retinal pathology that could interfere with interpretation (glaucoma, epiretinal membrane, diabetic macular edema, etc.). These exclusions could affect scans of one or both eyes.

A clinical, qualitative assessment of the OCT scans was performed independently by two research optometrists (BT and VS) to identify abnormal patterns of retinal thinning. Those with typical patterns of known ocular diseases such as glaucoma were excluded. Divergences were discussed and consensus was reached. The outcomes were classified as either (i) within normal limits/no obvious pattern, (ii) partial circular GC-IPL loss, (iii) advanced circular GC-IPL loss, (iv) partial temporal RNFL loss, (v) advanced temporal RNFL loss, or (vi) other abnormal pattern. These qualitative clinical assessments were used to group participants who presented abnormal patterns such as bilateral advanced GC-IPL circular loss, bilateral partial GC-IPL circular loss, bilateral advanced temporal RNFL loss, or bilateral partial temporal RNFL loss.

### 2.6. Study Population

The present study included persons who, at the time of the examinations, lived in or near Grassy Narrows and for whom there was an umbilical cord blood measurement in the existing database. A total of 91 community members fitted the criteria and 62 (65.9%) persons (29 men and 33 women) volunteered to undergo eye and vision examinations. OCT was performed on 59 persons. Following post hoc exclusions, both eyes could be analyzed for 57 participants and only one eye for 2 participants (two eyes were excluded due to measurement artefacts), for a total of 116 eyes (59 right and 57 left eyes).

Of the 59 participants who underwent eye and vision examinations, 49 (83.0%) likewise participated in an interviewer-administered general questionnaire. The questions were adapted from the First Nations Regional Health Survey (2008–2010) and covered demographics, employment, food security, lifestyle, and health status [64]. Participants also provided blood samples as part of the Niibin study. This subgroup served to test the possible contribution of covariates that may impact retinal thickness independently of Hg exposure: smoking status (non-smoker, former smoker, current smoker), frequency of alcohol consumption in the past 12 months, and glycated hemoglobin (HbA1c, as percentage of total hemoglobin).

### 2.7. Statistical Analyses

A series of descriptive analyses were conducted. Since many continuous variables were not normally distributed, group differences were assessed using non-parametric approaches: the Wilcoxon rank-sum test for two-group comparisons and the Kruskal–Wallis test for comparisons involving more than two groups. Categorical variables were compared in contingency tables using Pearson’s chi-squared test.

Given the strong inter-eye correlation (intraclass correlation coefficients > 0.82) for GC-IPL and RNFL thickness, the statistical power and precision of estimates were optimized by including data from both eyes whenever available [65]. This approach, which used ocular bilateral data, was further justified by the systemic nature of MeHg exposure, which is known to impact multiple components of the visual system, including the visual cortex [66,67], optic nerve [37], and retina [37,68].

A series of linear mixed-effects models (MEMs) were used to test the associations between umbilical cord blood Hg concentration and GC-IPL and RNFL thicknesses. Random intercepts at the participant level serve to capture within-subject correlations. This modeling enables the inclusion of clustered measures while representing the dependency structure inherent in the data. Fixed effects tested in the MEMs included umbilical cord blood Hg concentration and covariates such as age, sex, spherical refraction, best visual acuity, HbA1c, smoking status, and alcohol consumption. Participant ID was included as a random effect. A series of sensitivity analyses were conducted to validate the MEMs by repeating the analyses separately for each eye.

Logistic regression models were used to estimate the Odds Ratio of having clinically diagnosed bilateral abnormal retinal patterns.

The threshold of significance in all statistical analyses was set at *p* ≤ 0.05.

Database management and descriptive statistical analyses were assessed using JMP Professional 17.0 (2022 JMP Statistical Discovery LLC, Cary, NC, USA). For linear mixed-effects models and logistic regressions, a series of packages from R Statistical Software (version 2025.05.01+513; R Core Team, 2025, Vienna, Austria) were utilized (lme4, ggplot2 and redress, lmerTest, robustlmm, sandwich, performance, sjPlot, lmetest).

## 3. Results

The present analyses included 59 individuals (54% women, 46% men); the mean age was 40.5 years (SD = 4.8) and the median age was 41 years (IQR 38–45), ranging from 30 to 50 years. Among those who provided further information (*n* = 49), 33 (67.3%) were current smokers and 23 (46.9%) consumed alcoholic beverages less than a few times a month over the past year. Thirty-seven (75.5%) were working at the time of the study. HbA1c concentrations (*n* = 42) varied between 4.9% and 14.6% with a mean concentration of 6.7% (SD = 2.3) and median concentration of 5.7% (IQR 5.4–7.2).

Umbilical cord blood Hg concentrations for the 59 participants ranged from 1.1 µg/L to 160 µg/L, with a mean concentration of 12.7 µg/L (SD = 22.1) and a median concentration of 6.9 µg/L (IQR 3.1–13.7). In total, 33 persons (55.9%) had umbilical cord blood Hg concentrations above the United States Environmental Protection Agency guideline of 5.8 µ/L [69] and 25 measurements (42.4%) were above the Health Canada recommendation of 8 µg/L [70].

Table 1 shows the visual characteristics for all eyes (*N* = 116). The median distance visual acuity was 0.1 logMAR (Snellen equivalent 20/25) and the median refractive error was −0.75D, corresponding to mild myopia. A logMAR visual acuity of 0.00 corresponds to normal 20/20 vision, with increasing scores indicating reduced visual acuity. A refractive value closer to 0.00 reflects minimal refractive error and a lower likelihood of requiring optical correction. The more negative the refractive value, the greater the possibility that the eye’s axial length is increased with a higher risk for reduced retinal thickness [57,58].

Table 2 shows the distribution of retinal thicknesses for the optic disc cube scan, measuring the retinal nerve fiber layer (RNFL), and the macular cube scan, measuring the ganglion cell-inner plexiform layers (GC-IPL). Median numerical thickness values and the percentage of eyes within the age-adjusted normal range (green/white) are presented. Average RNFL thickness was in the abnormal range (yellow and red code) for 17% of eyes. Average GC-IPL thickness was classified in the abnormal thinner range (yellow and red code) for almost 30% of eyes. No significant differences in thickness were observed between men and women or between older (>41 years) and younger (≤41 years) persons for all RNFL and GC-IPL quadrants (Wilcoxon Kruskal–Wallis; *p* > 0.10) (Table A1 and Table A2).

MEMs were constructed for all retinal thickness measures with ID as a random effect and tested for the fixed variables umbilical cord blood Hg, age, sex, spherical refraction, visual acuity, smoking, alcohol consumption, and HbA1c level. Only umbilical cord blood Hg, spherical refraction, and, occasionally, visual acuity entered significantly into the models and were retained for the subsequent analyses when *p* < 0.2. Table 3 presents the MEM estimates for the associations between umbilical cord blood Hg and retinal thickness parameters with spherical refraction as a fixed effect and participant ID as a random effect. A clear negative association was observed between umbilical cord blood Hg concentration and average macular GC-IPL thickness across all quadrants, indicating a thinning of these layers with higher prenatal Hg exposure. Similar results were found for RNFL, with the exception of the nasal and superior quadrants.

A series of sensitivity analyses carried out on the right and left eyes separately showed similar results, with the exception of the inferior and temporal RNFL quadrant of the right eye that showed significant retinal thinning with higher cord blood Hg (Table A3 and Table A4).

**Figure 1:**
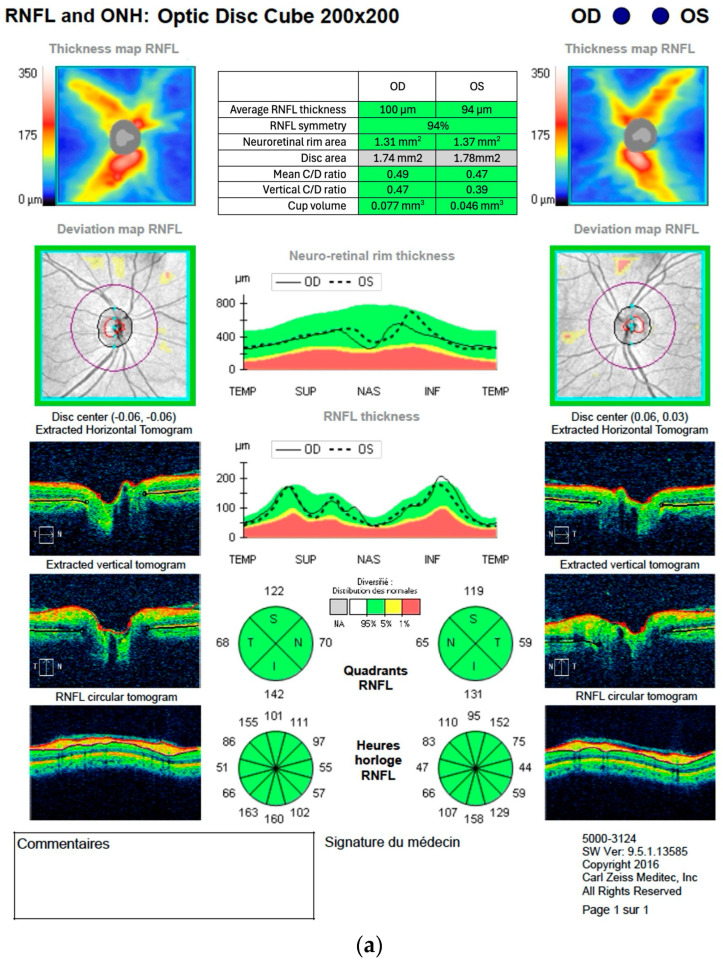

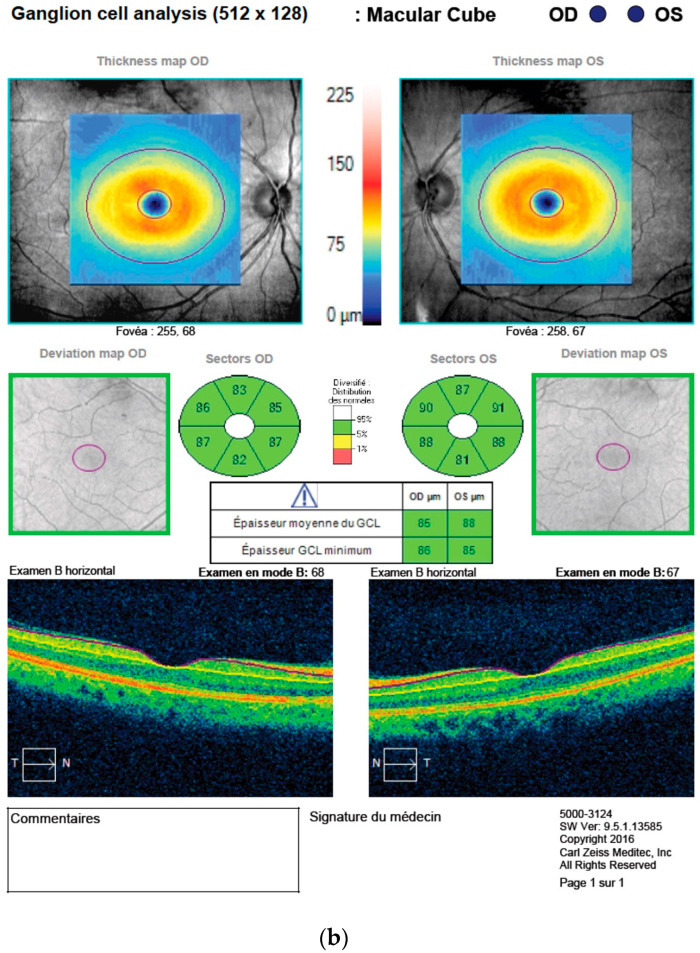
(**a**) Example of an optical coherence tomography scan report from the optic disc cube 200 × 200, showing a normative thickness and distribution of retinal nerve fiber layers of a 53-year-old participant. Certain labels in the figure were translated from French to English. (**b**) Example of an optical coherence tomography scan report from the macular cube 512 × 128, showing a normative thickness and distribution of macular ganglion cell and inner plexiform layers of a 53-year-old participant. Certain labels in the figures were translated from French to English and the table in (**a**) was rewritten in English, conserving original values and color codes.

**Figure 2:**
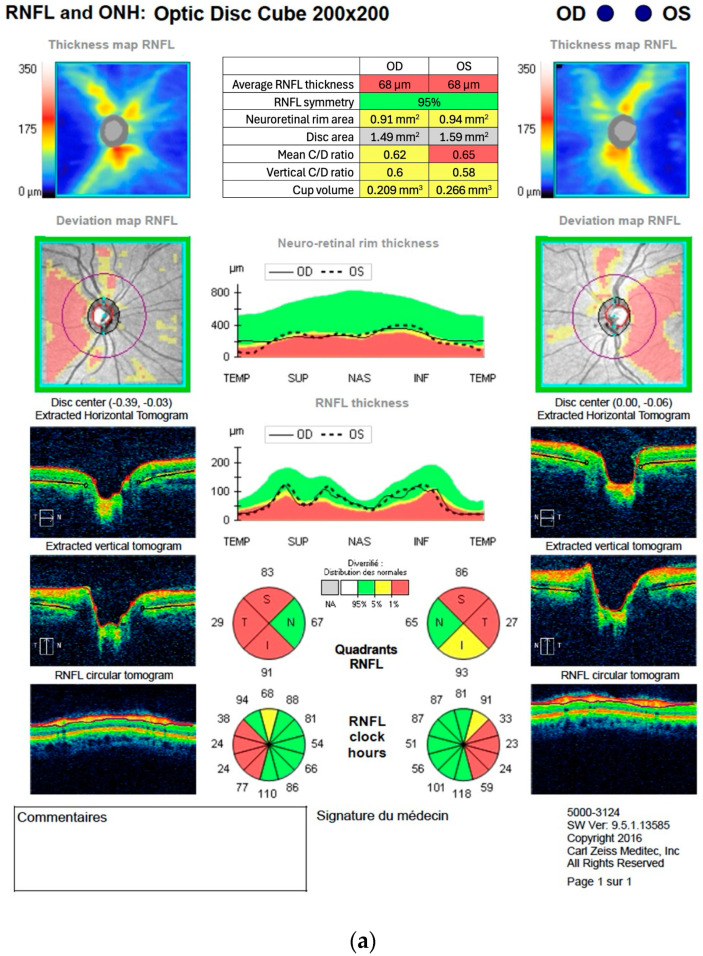

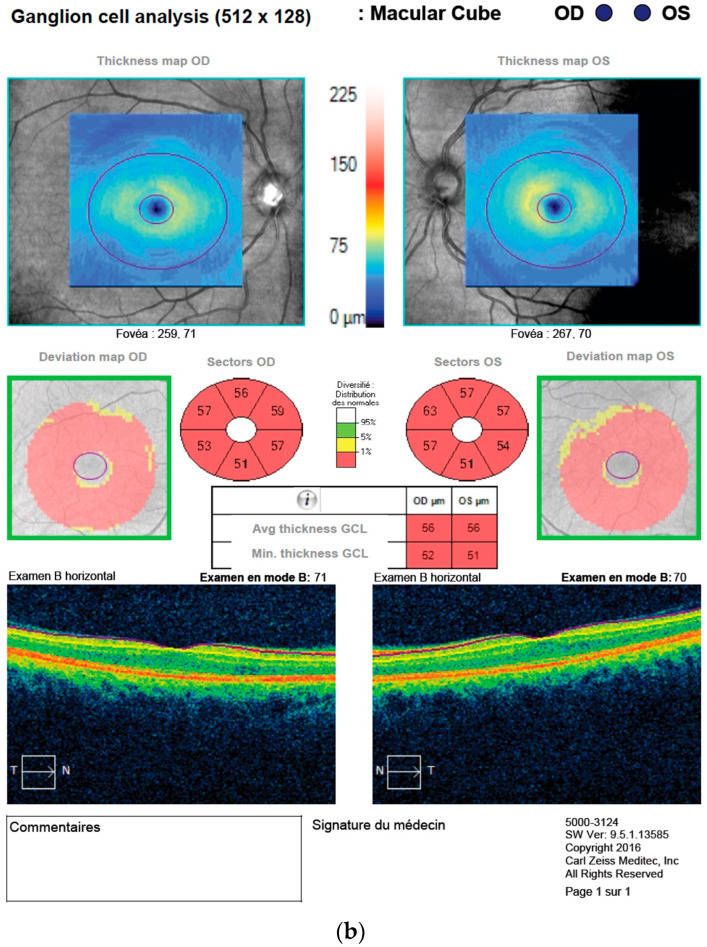
(**a**) Example of an optical coherence tomography scan report of a 38-year-old participant showing symmetric thinning of the temporal retinal nerve fiber layer on optic disc cube scan. Certain labels in the figure were translated from French to English. (**b**) Example of an optical coherence tomography scan report of a 38-year-old participant showing profound diffuse, bilateral ganglion cell and inner plexiform thinning on a macular cube scan. Certain labels in the figure were translated from French to English and the table in (**a**) was rewritten in English, conserving original values and color codes.

Figure 1 and Figure 2 are examples of OCT scans for the RNFL and GC-IPL thickness measurements. Figure 1a,b show scans of a 53-year-old participant whose results are considered within normal limits of the age-adjusted normative database, with no obvious pattern of retinal thinning. Figure 2a,b present the retinal thicknesses of a 38-year-old participant that were outside the normal limits according to the age-adjusted normative database. The thickness maps (Figure 2a) show advanced circular GC-IPL loss. The RNFL thickness maps (Figure 2b) reveal advanced temporal RNFL loss with notable symmetry between both eyes.

Qualitative clinical assessments showed that 20 participants (33.9%) presented either bilateral advanced GC-IPL circular loss, bilateral partial GC-IPL circular loss, bilateral advanced temporal RNFL loss, or bilateral partial temporal RNFL loss. Table 4 describes their general characteristics and type of loss. The median umbilical cord blood Hg concentration of those with abnormal qualitative clinical loss was significatively higher compared to the others (10.05 µg/L [IQR: 5.50–34.18] and 5.1 µg/L [IQR: 2.60–8.60], respectively; Wilcoxon chi-square = 7.94; *p* = 0.005). The odds of qualitative abnormal retinal thinning among individuals with umbilical cord blood Hg concentrations ≥ 5.8 µg/L was more than three times higher than those with lower levels (OR = 3.50; 95% CI = 1.11–12.52; *p* = 0.031). Neither age nor spherical refraction entered significantly into the logistic regression model or modified the beta estimate by more than 10%.

## 4. Discussion

This is the first study, to our knowledge, to show a positive association between prenatal MeHg exposure and retinal thinning assessed in adulthood. The most prominent finding was a reduction in retinal thickness across all macula sectors of the GC-IPL layers and some regions of the RNFL layers, namely, the average, inferior, and superior sectors.

In the present study, thinning observed across all quadrants of the macular region may reflect a potential impact of MeHg on retinogenesis. To date, few studies have investigated the effects of MeHg in an embryonic context. Research using zebrafish larvae models has demonstrated alterations in retinal gene expression associated with a loss of GABAergic neurons in the GC-IPL [47,49]. This neuronal loss, combined with apoptosis within the inner nuclear layer and known structural effects on retinal photoreceptors, may collectively contribute to the development of visual impairments [49]. A study of chicken embryos reported disrupted organization of ganglion cells during development; the authors suggest that the embryonic retina is more vulnerable to MeHg exposure than the adult retina during adulthood [71]. In humans, the macula contains almost 50% of the retinal ganglion cells and is crucial for many visual functions [72]. The macular ganglion cell layer thinning observed in the present study with respect to prenatal MeHg exposure resembles changes reported in studies of other conditions such as preperimetric glaucoma, multiple sclerosis, and Alzheimer’s disease [73,74,75,76,77,78]. Regarding the effects of MeHg exposure to other macular layers, such as the photoreceptors, further studies are warranted, as these analyses were not possible in the current study.

In the present study, the average RNFL layer also exhibited an inverse association between thickness and increasing umbilical cord blood Hg concentrations, although to a lesser extent. While some animal studies suggest the accumulation of inorganic Hg in the optic nerve during the prenatal period [79,80], no definitive association with MeHg has been established in humans. As the measurement of the RNFL scan captures the structure of retinal fibers originating from the entire retinal surface as they converge into the optic nerve, the lesser thinning observed here, compared to the macular area, could reflect the fact that MeHg may not be deposited equally everywhere in the retina. MeHg may have a stronger affinity for the macula, which is very active metabolically, with a higher density of retinal ganglion cells in the parafoveal region [72,81,82].

The clinical qualitative evaluation of the OCT thinning patterns revealed that one-third of participants showed clinically significant and abnormal bilateral loss of retinal ganglion cell thickness, either a circular macular pattern of GC-IPL loss or diffuse temporal loss on RNFL. Those with umbilical cord blood Hg concentrations over 5.8 µg/L, the United States Environmental Protection Agency (EPA) guidelines [69], had over a three-fold likelihood of abnormal retinal thinning patterns [70]. With a median age of 40, these individuals are younger than those typically presenting retinal patterns from ocular pathologies, such as glaucoma or other optic neuropathies [83,84]. Differentiating abnormal retinal thinning patterns is crucial to prevent false diagnoses of glaucoma, as the rate of misdiagnosis from suspicious retinal ganglion cell thinning can reach up to 40% [85]. Glaucoma is characterized by a gradual asymmetric loss of retinal ganglion cells, often starting at the superior or inferior fibers of the macula, preceding the typical arcuate visual field defect [17,86]. Inflammatory neuropathies, like optic neuritis, are typically unilateral and present with a sudden onset of vision loss [87]. Both arteritic and non-arteritic ischemic optic neuropathies are also commonly unilateral and rarely cross the horizontal midline of both visual field and GC-IPL layers [87,88].

MeHg prenatal exposure has been associated with loss of other visual functions. In monkeys, it is associated with lower contrast sensitivity in later life [89]. Although studies of children did not observe associations with prenatal MeHg exposure [90,91], near visual contrast sensitivity loss and acquired dyschromatopsia have both been associated with current adult Hg exposure in fish-eating communities [92,93,94].

The observation of bilateral consistency of the retinal findings supports a hypothesis in which MeHg may act broadly across the visual system, potentially through mechanisms involving central neurodevelopmental pathways. It is plausible that MeHg reaches and affects both developing retinas similarly, contributing to a broad effect on retinal structure. However, in the absence of clear mechanistic evidence, this does not exclude potential concurrent focal toxic effects.

Among the strengths of this study is the innovative use of OCT technology. To date, OCT has only been used in studies to assess potential retinal neurotoxicity associated with inorganic Hg [95,96,97]. To our knowledge, this is the first study to document and explore the impact of prenatal MeHg exposure on human retinal architecture. A further strength is its inclusion in the Niibin study that employed a broad transdisciplinary, participatory approach, integrating multiple domains and comprehensive general questionnaires.

There are several limitations to the study. Recruitment was based on existing measures of umbilical cord blood Hg and few persons in this group had sufficient biomarkers of long-term exposure measurement to analyze the possible contribution of postnatal exposure. Although the exposure variable temporally precedes the outcome, retinal thickness was only measured once, which limits causal inference. Retinal thickness loss measured in adulthood may represent MeHg fetal programming [98] or the combined effects of prenatal Hg and subsequent postnatal exposure over the lifetime [98]. It would be valuable for future studies to examine whether long-term MeHg exposure impacts retinal thickness.

In the present study, only the inner retinal thickness (GC-IPL) was measured. This may have limited certain interpretations. A study on zebrafish larva models has shown that there is a possible accumulation of MeHg in the outer layers of the retina, more specifically in the photoreceptors [99]. While thinning of the inner retina was associated with prenatal MeHg exposure, a global portrait of the retinal architecture would provide additional information, particularly on the layers that are responsible for visual acuity and color vision. Although we accounted for the major known confounding factors, other confounding factors may be present and might not have been captured by our analyses. Additionally, minimal information was available for postnatal health and lifestyle. We did, however, account for several possible contributing factors, assessed at the time of the examination and known to affect retinal thickness; none influenced the relation between prenatal Hg exposure and retinal thickness. Interestingly, despite the absence of data on postnatal exposure in this population, which was not necessarily constant over time, the association between prenatal exposure and retinal thickness was significant. Hg umbilical cord blood concentrations reflect exposure throughout pregnancy and fetal neurodevelopment is known to be very vulnerable to methylmercury exposure [100,101]. Our analyses must be considered as an estimation of the contribution of prenatal Hg exposure to later-life outcomes rather than an attempt to isolate a pure mechanistic effect.

## 5. Conclusions

These results show that higher prenatal exposure to MeHg was associated with greater retinal thinning in adulthood, adding to the burden of the MeHg-related health impacts on Grassy Narrows First Nation. These findings highlight the importance of assessing retinal thickness using OCT in the diagnosis and monitoring of MeHg-related visual limitations and the need for appropriate and accessible eye care for populations with documented or suspected MeHg exposure. Longitudinal OCT studies would be needed to explore how the type of retinal thinning observed evolves over time. These findings reveal impacts that extend beyond established national guidelines and highlight the need for reassessing preventive measures regarding environmental contaminant control.

## Figures and Tables

**Table 1:** Visual characteristics of all eyes (*N* = 116).

Visual Characteristics	Mean (SD)	Median (IQR)	Min–Max
Best visual acuity with pinhole (logMAR)	0.15 (0.16)	0.1 (0.1–0.2)	−0.1–0.8
Spherical refraction (diopters)	−0.94 (1.73)	−0.75 (−1.75–+0.25)	−7.50–+2.50

Abbreviations: IQR: interquartile range, logMAR: logarithm of minimum angle of resolution.

**Table 2:** Distribution of retinal layer thicknesses measured by optical coherence tomography (OCT).

	Median Thickness μm (IQR)	Retinal Thicknesses Within Normal Range (Green/White) *n* (%)
**Optic disc cube**	*n* = 113 ^a^	*n* = 106 ^c^
Average RNFL thickness	91 (83–100)	88 (83%)
Thickness (by quadrant)		
Superior	113 (103–123)	89 (84%)
Nasal	71 (64–80)	104 (98.1%)
Inferior	121 (111–134)	97 (91.5%)
Temporal	55 (50–61)	89 (84%)
**Macular cube**	*n* = 113 ^b^	*n* = 109 ^d^
Average GC-IPL thickness	80 (74–84)	78 (71.6%)
Thickness (by section)		
Superior	80 (77–86)	84 (77.1%)
Superior nasal	83 (77–86)	86 (78.9%)
Inferior nasal	80 (74–85)	87 (79.8%)
Inferior	76 (71–80)	82 (75.2%)
Inferior temporal	79 (73–83)	80 (73.4%)
Superior temporal	79 (74–83)	80 (73.3%)

Abbreviations: RNFL: retinal nerve fiber layer, GC-IPL: ganglion cell and inner-plexiform layers, IQR: interquartile range, Green/White: thickness value thicker or within normal range compared to database. ^a^: Three eyes were removed from the analysis of RNFL thickness after post hoc exclusions. ^b^: Three eyes were removed from the analysis of GC-IPL thickness after post hoc exclusions. ^c^: Ten eyes were removed from the analysis of color-coded RNFL thickness after post hoc exclusions. ^d^: Seven eyes were removed from the analysis of color-coded GC-IPL thickness after post hoc exclusions.

**Table 3:** Linear mixed effects model estimates for umbilical cord Hg for measures of retinal ganglion cell-inner plexiform layer (GC-IPL) and retinal nerve fiber layer (RNFL) thickness outcomes.

OCT Measures (y Variable)	*N*	Estimate (µg/L)	95% CI	*p*-Value
**Macular cube**				
Average GC-IPL thickness	109	−0.32	−0.49–−0.15	<0.001
Thickness (by section)				
Superior	110	−0.18	−0.27–−0.09	<0.001
Superior nasal	111	−0.37	−0.55–−0.19	<0.001
Inferior nasal	111	−0.34	−0.52–−0.16	<0.001
Inferior	111	−0.32	−0.49–−0.14	0.001
Inferior temporal	111	−0.31	−0.51–−0.11	0.003
Superior temporal	111	−0.31	−0.47–−0.14	<0.001
**Optic disc cube**				
Average RNFL thickness	109	−0.28	−0.56–−0.01	0.047
Thickness (by quadrant)				
Superior	109	−0.26	−0.62–−0.09	0.146
Nasal	111	−0.11	−0.40–0.17	0.437
Inferior	110	−0.46	−0.87–−0.05	0.028
Temporal	111	−0.21	−0.42–0.01	0.064

Abbreviations: CI: confidence interval, RNFL: retinal nerve fiber layer, GC-IPL: ganglion cell and inner-plexiform layers.

**Table 4:** Retinal thinning distribution and general characteristics with abnormal qualitative clinical assessment of OCT scans.

	*N*	*n* (%)	Mean	Median (IQR)	Min–Max
**Clinical patterns of retinal thinning loss**	20				
Bilateral partial GC-IPL circular loss	8	8 (40.0%)			
Bilateral advanced GC-IPL circular loss	12	12 (60.0%)			
Bilateral partial or advanced temporal RNFL loss	8	8 (35.0%)			
**Characteristics of subgroup with abnormal results**					
Age at time of visual examination (years)	20		40	41 (38–45)	30–50
Sex (woman)	20	9 (45.0%)			
Best visual acuity with pinhole (logMAR)	20		0.2	0.2 (0.1–0.2)	−0.1–0.6
Umbilical cord blood Hg concentration (µg/L)	20		23.7	10.1 (5.5–34.2)	2.3–160

Abbreviations: IQR: interquartile range, RNFL: retinal nerve fiber layer, GC-IPL: ganglion cell and inner-plexiform layers.

## Data Availability

Restrictions apply over these data. In respect of the principles of OCAP (Ownership, Control, Access, and Possession), the data are the property of the Grassy Narrows First Nation.

## Abbreviations

The following abbreviations are used in this manuscript:

GC-IPL	Ganglion cell-inner plexiform layer
RNFL	Retinal nerve fiber layer
OCT	Optical coherence tomography
Hg	Mercury
MeHg	Methylmercury
CNS	Central nervous system
VEP	Visual evoked potential
MEM	Mixed-effect model

## Appendix A

**Table A1:** GC-IPL thickness with respect to age category and sex.

	Age			Sex		
	≤41 Years	>41 Years		Women	Men	
	(*n* = 60)	(*n* = 53)		(*n* = 64)	(*n* = 49)	
GC-IPL Thickness	Median (IQR) (µm)		*p*-Value *	Median (IQR) (µm)		*p*-Value *
Average	80 (74–85)	78 (73–82)	0.208	79 (74–83)	80 (74–84)	0.560
Sectoral						
Superior	82 (75–86)	80 (75–84)	0.385	81 (76–85)	80 (74–86)	0.848
Superior nasal	84 (78–89)	82 (75–86)	0.104	82 (76–86)	84 (78–89)	0.265
Inferior nasal	81 (74–86)	80 (74–83)	0.332	81 (74–88)	78 (74–84)	0.183
Inferior	76 (71–81)	76 (71–80)	0.717	76 (70–81)	76 (71–80)	0.783
Inferior temporal	79 (73–84)	78 (72–82)	0.232	79 (73–84)	79 (73–83)	0.636
Superior temporal	79 (73–85)	78 (74–81)	0.464	79 (73–86)	79 (74–82)	0.810

Abbreviation: GC-IPL: ganglion cell and inner plexiform layer, IQR: interquartile range. * Non parametric Wilcoxon Kruskal–Wallis test.

**Table A2:** RNFL thickness with respect to age category and sex.

	Age			Sex		
	≤41 Years	>41 Years		Women	Men	
	(*n* = 58)	(*n* = 56)		(*n* = 61)	(*n* = 52)	
RNFL Thickness	Median (IQR) (µm)		*p*-Value *	Median (IQR) (µm)		*p*-Value *
Average	92 (81–101)	91 (84–97)	0.893	91 (82–100)	91.5 (84–100)	1.000
Sectoral						
Superior	115 (98–123)	112 (106–122)	0.838	114 (103–123)	113 (102–123)	0.511
Nasal	72 (64–83)	71 (64–76)	0.631	70 (64–79)	73 (64–81)	0.562
Inferior	121 (110–136)	121 (111–132)	0.986	120 (111–136)	122 (109–133)	0.749
Temporal	56 (50–60)	55 (50–62)	0.809	55 (49–61)	56 (51–62)	0.809

Abbreviation: RNFL: retinal nerve fiber layer, IQR: interquartile range. * Non parametric Wilcoxon Kruskal–Wallis test.

**Table A3:** General linear model (GLM) for umbilical cord blood Hg concentration estimates (µg/L) with respect to retinal thickness in the right eye (OD).

	*n*	Hg (µg/L) Estimate	95% CI	*p*-Value
**RNFL thickness**				
Average	55	−0.30	−0.54–0.01	0.056
Sectoral thicknesses				
Superior	56	−0.23	−0.63–0.17	0.260
Nasal	55	−0.01	−0.39–−0.25	0.647
Inferior	46	−0.55	−0.99–0.10	0.017
Temporal	45	−0.22	−0.44–0.00	0.047
**GC-IPL thickness**				
Average	54	−0.36	−0.55–−0.20	<0.001
Sectoral thicknesses				
Superior	54	−0.39	−0.57–−0.22	<0.001
Superior nasal	55	−0.39	−0.56–−0.21	<0.001
Inferior nasal	46	−0.40	−0.59–−0.20	<0.001
Inferior	55	−0.31	−0.49–−0.13	0.001
Inferior temporal	54	−0.33	−0.53–−0.12	<0.001
Superior temporal	54	−0.36	−0.53–−0.19	<0.001

Abbreviations: CI: confidence interval, RNFL: retinal nerve fiber layer, GC-IPL: ganglion cell inner plexiform layer. Mixed-effect models included spherical refraction and best visual acuity.

**Table A4:** General linear model (GLM) umbilical cord blood Hg concentration estimates (µg/L) with respect to retinal thickness in the left eye (OS).

	*n*	Hg (µg/L) Estimate	95% CI	*p*-Value
**RNFL**				
Average	52	−0.32	−0.56–−0.08	0.010
Sectoral thicknesses				
Superior	53	−0.27	−0.67–−0.13	0.180
Nasal	55	−0.11	−0.40–0.17	0.452
Inferior	54	−0.42	−0.81–−0.03	0.035
Temporal	54	−0.19	−0.41–0.03	0.096
**GC-IPL**				
Average	54	−0.33	−0.48–−0.19	<0.001
Sectoral thicknesses				
Superior	54	−0.34	−0.50–−0.18	<0.001
Superior nasal	54	−0.36	−0.52–−0.20	<0.001
Inferior nasal	53	−0.33	−0.50–−0.16	<0.001
Inferior	53	−0.32	−0.46–−0.17	<0.001
Inferior temporal	53	−0.34	−0.50–−0.19	<0.001
Superior temporal	54	−0.29	−0.43–−0.15	<0.001

Abbreviations: CI: confidence interval, RNFL: retinal nerve fiber layer, GC-IPL: ganglion cell inner plexiform layer. Mixed-effect models included spherical refraction and best visual acuity.
